# Mitochondrial control through nutritionally regulated global histone H3 lysine-4 demethylation

**DOI:** 10.1038/srep37942

**Published:** 2016-11-29

**Authors:** Maria Soloveychik, Mengshu Xu, Olga Zaslaver, Kwanyin Lee, Ashrut Narula, River Jiang, Adam P. Rosebrock, Amy A. Caudy, Marc D. Meneghini

**Affiliations:** 1 Department of Molecular Genetics, University of Toronto, ON, M5S 1A8, Canada; 2 Terrence Donnelly Centre for Cellular & Biomolecular Research, University of Toronto, ON, M5S 3E1, Canada

## Abstract

Histone demethylation by Jumonji-family proteins is coupled with the decarboxylation of α-ketoglutarate (αKG) to yield succinate, prompting hypotheses that their activities are responsive to levels of these metabolites in the cell. Consistent with this paradigm we show here that the *Saccharomyces cerevisiae* Jumonji demethylase Jhd2 opposes the accumulation of H3K4me3 in fermenting cells only when they are nutritionally manipulated to contain an elevated αKG/succinate ratio. We also find that Jhd2 opposes H3K4me3 in respiratory cells that do not exhibit such an elevated αKG/succinate ratio. While *jhd2∆* caused only limited gene expression defects in fermenting cells, transcript profiling and physiological measurements show that *JHD2* restricts mitochondrial respiratory capacity in cells grown in non-fermentable carbon in an H3K4me-dependent manner. In association with these phenotypes, we find that *JHD2* limits yeast proliferative capacity under physiologically challenging conditions as measured by both replicative lifespan and colony growth on non-fermentable carbon. *JHD2*’s impact on nutrient response may reflect an ancestral role of its gene family in mediating mitochondrial regulation.

As with all other histone lysine methylations, methylation of histone H3 on lysine-4 exists in mono, di, or tri methylated (H3K4me1, 2, 3) forms, and these modification states control differing mechanistic outputs through the recruitment of H3K4me-specific binding proteins. Significant advancements in our understanding of H3K4me function have come from organisms ranging from unicellular yeasts to humans. These studies have revealed that H3K4me is associated with actively transcribed chromatin where it impacts gene expression through diverse mechanisms. Unique among model organisms, yeast possesses solitary H3K4 methyltransferase and demethylase enzymes. These enzymes, encoded by *SET1* and *JHD2*, are orthologous to the mammalian MLL and JARID1 families, respectively. Curiously, deletion of *JHD2* results in imperceptible phenotypic consequence in cells grown using standard laboratory conditions, impeding the utilization of yeast as a model system to study this conserved chromatin regulator^1^,^2^.

Jhd2 belongs to an expansive protein family distinguished by the presence of a JmjC domain. The JmjC domain, initially identified in the C-terminal region of the mouse Jumonji protein, is now known to mediate the demethylation of histone lysine residues^3^,^4^. Histone demethylation by JmjC domain containing proteins *in vitro* requires αKG, which is converted to succinate in the demethylation reaction^4^. Subsequent studies have suggested that succinate accumulation can inhibit *in vivo* histone demethylation by JmjC domain proteins^5^,^6^,^7^. These findings have prompted the hypothesis that histone demethylation by JmjC proteins may be responsive to cellular metabolic state^8^. This hypothesis has received support from studies in embryonic stem (ES) cells, where nutritional conditions leading to an elevated αKG/succinate ratio were associated with UTX- and JMJD3-dependent reductions in levels of H3K27me3^9^. Curiously, although multiple histone lysine residues were hypo-methylated in response to increased αKG/succinate in ES cells, H3K4me3 was unperturbed^9^. Among the many possible explanations for this incongruity is that JmjC enzymes controlling H3K4 demethylation may be varyingly responsive to αKG levels and/or competitive succinate inhibition *in vivo*. A more recent study demonstrated reductions in H3K4me3 levels in response to genetic conditions causing elevated αKG, though it was not clear if this was through H3K4 demethylation^10^.

The JARID1 family comprises the only known JmjC domain containing demethylases with specificity for H3K4. Using nutritional manipulation of yeast, here we address the regulation both of and by the JARID1-family H3K4 demethylase Jhd2, and provide evidence for its particular role in restraining mitochondrial respiration. Corroborating the prevailing view concerning metabolic regulation of JmjC demethylases, we observe *JHD2*-dependent bulk H3K4me3 reductions in fermenting cells that are nutritionally manipulated to elevate the αKG/succinate ratio. Somewhat surprisingly, *JHD2* exerts a limited impact on mRNA accumulation in these cells. We also observe *JHD2*-dependent reductions in H3K4me3 levels in cells grown on nonfermentable carbon that occur in the absence of an elevated ratio of αKG/succinate. Unlike in fermenting cells, these H3K4me3 alterations are associated with widespread gene expression defects with a particular impact on the abundance of mRNAs that encode mitochondrial proteins. Prompted by our gene expression profiling results, we interrogated mitochondrial function and provide evidence that *JHD2* restrains mitochondrial respiration through H3K4 demethylation. These gene expression and physiological phenotypes are associated with improved proliferative capacity of *jhd2∆* cells in replicative lifespan experiments or colony growth in nonfermentable carbon.

## Results

### *JHD2* restrains mitochondrial respiration in cells grown using non-fermentable carbon

**Figure 1:**
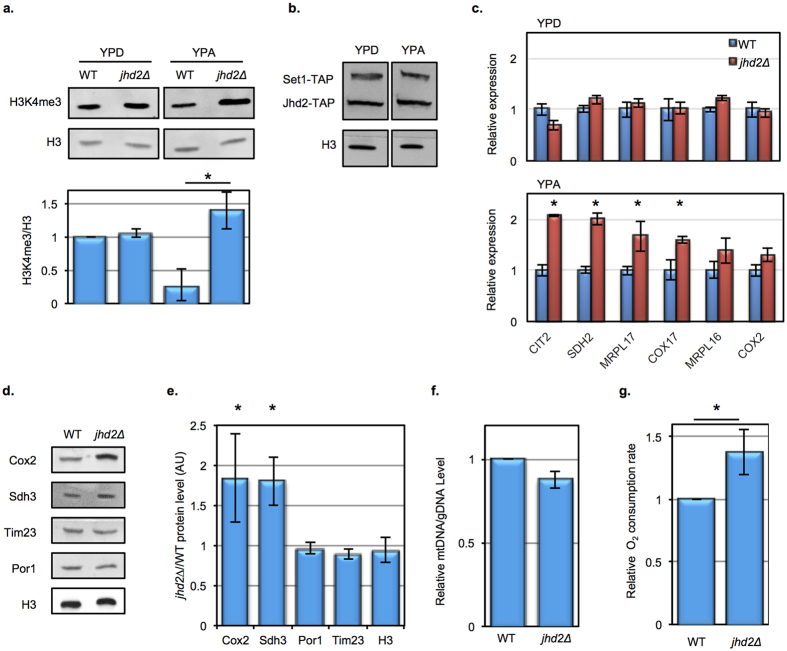
*JHD2* restrains respiration in nonfermentable growth conditions. (**a**) H3K4me3 and pan-H3 abundance in WT (MSY723) and *jhd2Δ* (MSY724) cells grown in the indicated media was measured using western blotting. The lower panel shows H3K4me3/H3 quantification for n = 3 normalized to WT in YPD with error bars depicting 1 standard deviation (s.d.). Significance as calculated by a two-tailed t-test is shown where *p = 0.006. (**b**) Western blot analysis of WT cells is shown with growth media indicated. Jhd2 and Set1 levels were determined using endogenous TAP tags using the strain MSY1074. (**c**) WT (MSY723) and *jhd2∆* (MSY724) cells were grown in YPD or YPA, followed by RT-qPCR quantification of the indicated transcripts. n = 4, and error bars reflecting 1 s.d. are shown. Significance as calculated by a two-tailed t-test is shown where *p = 0.02 (*CIT2*), 0.006 (*SDH2*), 0.04 (*MRPL17*), and 0.05 (*COX17*). (**d**) WT and *jhd2∆* cells were grown in YPA and extracts were analyzed by western blots probing for the indicated proteins. (**e**) The relative protein levels for 3 biological replicates with 1 s.d. error bars is shown where *p = 0.05 (Cox2) and 0.01 (Sdh3). (**f**) DNA was extracted from the same cells and relative levels of mtDNA (*COX2*) to a genomic locus (*ECM5*) were determined by qPCR. (**g**) Oxygen consumption rates were measured in WT and *jhd2∆* strains grown in YPA, n = 3, 1 s.d. is shown. Significance as calculated by a two-tailed t-test is shown where *p = 0.02.

Although Jhd2 has been confirmed as a histone demethylase with specificity for H3K4 *in vitro*, several studies have shown that *jhd2∆* has no detectable consequence for bulk H3K4me levels or relative gene expression in cells grown in rich (YP) media containing glucose as the sole carbon source (YPD)^1^,^2^,^11^,^12^,^13^. We previously demonstrated that *JHD2* globally impacts gene expression and H3K4me3 during sporulation, which occurs in nitrogen-starved cells in the presence of the non-fermentable carbon source acetate^2^. We therefore considered that mitotically proliferating cells grown using acetate might also exhibit *jhd2∆* phenotypes. To test this, we used western blotting to measure bulk H3K4me3 levels in cells grown in YPD or in rich media with acetate as the sole carbon source (YPA). In agreement with previous studies^2^,^11^, we detected no differences in bulk H3K4me3 levels from wild type (WT) and *jhd2∆* strains grown in YPD (Fig. 1a). In WT cells grown in YPA, we found that bulk H3K4me3 was markedly decreased and that *JHD2* was required for this nutrient specified H3K4me3 reduction (Fig. 1a). This effect of *jhd2∆* was not observed for methylation of histone H3 on lysine-36, the only other known histone target of demethylation in yeast (Supplementary Data Fig. 1). As the protein levels of Jhd2 and Set1 were unchanged in these conditions (Fig. 1b), these results suggest that an increase in Jhd2 activity caused H3K4me3 demethylation in this obligate respiratory context. Of the five JmjC domain proteins encoded by the budding yeast genome (Jhd2, Ecm5, Gis1, Rph1, and Jhd1), we detected a bulk H3K4me3 defect only in *jhd2∆* cells grown in YPA (data not shown), consistent with biochemical and phylogenetic studies suggesting that Jhd2 is the only yeast Jumonji protein with specificity for H3K4me3^12^,^13^,^14^.

H3K4me3 is associated with actively transcribed chromatin, where it impacts gene expression through diverse mechanisms. We interrogated YPA grown cells for global gene expression consequences of *jhd2∆* using microarray transcript profiling. In contrast to the negligible role of *JHD2* in YPD grown cells^1^,^2^,^11^, *jhd2∆* mutants grown in YPA exhibited significant alterations in transcript abundance for 157 genes (Supplementary Table 1). Although the number of *JHD2*-regulated transcripts in YPA was relatively small, gene ontology (GO) analysis revealed that the 105 mRNAs that were *JHD2*-repressed were highly enriched for those encoding mitochondrial proteins, particularly those that comprise the electron transport chain (ETC) (*p* < 2e-13, Supplementary Table 1). Quantitative PCR (qPCR) of reverse transcribed RNA from independent biological replicates confirmed that nuclear encoded mRNAs specifying mitochondrial proteins were increased in abundance in *jhd2∆* cells grown in YPA media, but not in YPD media (Fig. 1c).

Owing to their release from glucose mitochondrial repression (discussed below), cells grown in YPA accumulated mitochondrial biomass and exhibited elevated respiratory capacity compared with YPD (Supplementary Data Fig. 2). We used western blotting to determine if the increased transcript levels for mitochondrial ETC components in *jhd2∆* mutants grown in YPA were associated with increased levels of ETC proteins. In YPA media, we found that *jhd2∆* caused increased accumulation of the ETC components Cox2 and Sdh3, but not of the mitochondrial membrane proteins Por1 and Tim23 (Fig. 1d and e). Although we didn’t evaluate *SDH3* mRNA levels in our RT-qPCR experiments, our microarray data showed *SDH3* mRNA to be almost 2-fold upregulated in *jhd2∆* (Supplementary Table 1). Moreover, both *TIM23* and *POR1* mRNA were unchanged in *jhd2∆* in this data set (Supplementary Table 1). Quantification of mitochondrial gDNA and live cell fluorescence imaging experiments showed that the abundance of mitochondria was not increased in *jhd2∆* cells grown in YPA (Fig. 1f and data not shown). Together with our transcript profiling data, these observations suggest that *jhd2∆* caused increased ETC content of mitochondria in YPA rather than an increased abundance of cellular mitochondria *per se*. As *COX2* is encoded in the mitochondrial genome and *COX2* transcript levels were not significantly increased in *jhd2∆* (Fig. 1c), we speculate that *JHD2* impacts the translation of mitochondrial-encoded mRNAs and/or the stability of their respective proteins through mechanisms that are independent of its role in controlling the accumulation of nuclear encoded mRNAs.

Because the ETC contributes to mitochondrial respiration, our findings suggested that *jhd2∆* might exhibit a respiratory phenotype. To test this, we measured the rate of dissolved oxygen consumption in WT and *jhd2∆* cells and found that, in accordance with its gene expression defects, *jhd2∆* caused increased basal mitochondrial respiration in YPA grown cells (Fig. 1g). To confirm that the oxygen consumption we measured was attributable to mitochondrial respiration, we treated cells with the mitochondrial poison potassium cyanide (KCN), which inhibits mitochondrial oxygen consumption by interfering with cytochrome oxidase activity, or with the mitochondrial uncoupler CCCP, which stimulates maximal respiration. Treatment of cells with CCCP caused increased oxygen consumption while KCN abolished it (Supplementary Data Fig. 3). We routinely included these controls in our experiments and always observed CCCP induced maximal oxygen consumption rates that mirrored basal rates and abolishment of oxygen consumption in KCN treated cells, confirming that the oxygen consumption rates we measured were due to mitochondrial respiration.

### Metabolic cues impact *JHD2* function

Although histone demethylation by JmjC proteins requires αKG, numerous studies suggest that their *in vivo* activities are impacted not only by αKG accumulation, but also by other metabolites that can inhibit their enzymatic activity. Of particular significance is succinate, a product of the demethyation reaction that competitively inhibits JmjC activity *in vitro*, and whose accumulation has been correlated with decreased JmjC-mediated histone demethylation *in vivo*^5^,^6^,^7^. Indeed, findings from ES cells suggest that *in vivo* activity of JmjC enzymes reflects the ratio of αKG/succinate rather than the accumulation of αKG *per se*^9^. To interrogate the relationship between *JHD2* function and metabolite accumulation in the cell, we engineered prototrophic strains that enable growth in synthetic Y media with no amino acid supplementation, enabling precise control of carbon and nitrogen sources and measurement of metabolite abundance using liquid chromatography mass spectrometry (LC-MS).

**Figure 2:**
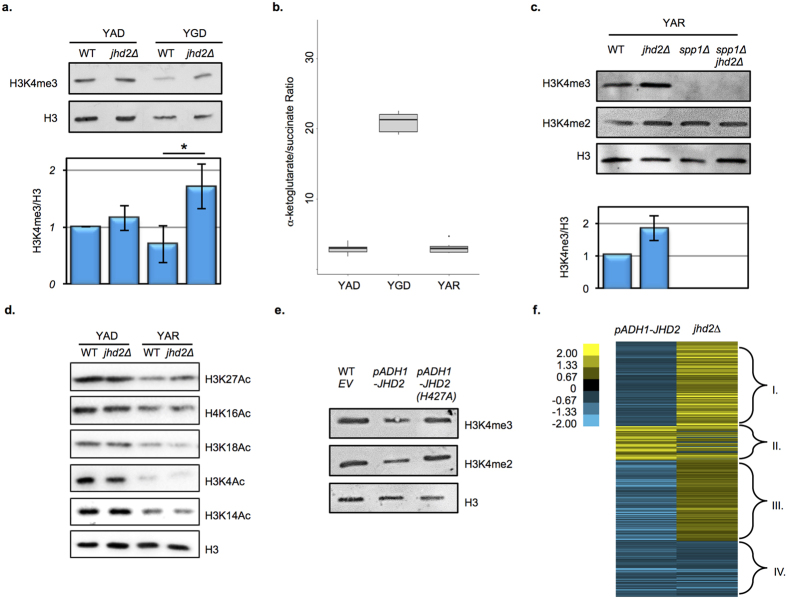
Nutritional conditions impact Jhd2 control of H3K4me3 and gene expression. (**a**) H3K4me3 and pan-H3 abundance in WT (MSY723) and *jhd2Δ* (MSY724) cells grown in the indicated media was measured using western blots. The lower panel shows H3K4me3/H3 quantification for n = 3 with 1 s.d. shown. Significance as calculated by a two-tailed t-test is shown where *p = 0.02. (**b**) The ratio of intracellular αKG/succinate levels was determined in prototrophic wild type cells (RCY308) grown in the indicated media. The boxes represents the upper and lower quartiles separated by the median for 6 biological replicates. Whiskers indicate the maxima and minima of the data. (**c**) Western blot analysis of H3K4me3, H3K4me2, and pan-H3 levels in the indicated genotypes grown in YAR (MSY723, MSY724, MSY840, MSY838). The lower panel shows H3K4me/H3 quantification for n = 3 normalized to WT controls with 1 s.d. error bars. Significance as calculated by a two-tailed student’s t-test is shown where *p = 0.08. (**d**) Western blot analysis of H3K27Ac, H3K16Ac, H3K18Ac, H3K4Ac, H4K16Ac, and pan-H3 levels in the indicated genotypes (MSY723, MSY724) grown in YAD or YAR is shown. (**e**) Western blot detection of H3K4me3, H3K4me2, and pan-H3 is shown from extracts of cells grown in YAR and containing empty vector control (MMY5087), ***P***_*ADH1*_*-JHD2* (MMY5086) or ***P***_*ADH1*_*-JHD2-H427A* (MMY5085) encoding plasmids is shown. (**f**) Genes whose respective mRNAs were significantly altered in abundance in both *JHD2*-overexpressing cells and *jhd2∆* grown in YAR were assembled into 4 groups using k-means clustering. A heat map indicating relative changes using a Log_2_ scale is shown, with respective k-means groups bracketed on the right.

As in YPD, fermenting *jhd2∆* cells grown in Y media with ammonium and glucose as the sole sources of nitrogen and carbon (YAD) did not exhibit a significant increase in bulk H3K4me3 levels compared with WT (Fig. 2a). We hypothesized that the absence of any detectable H3K4 demethylation by Jhd2 in these conditions was due to insufficient levels of αKG in the cells. To nutritionally manipulate intracellular αKG levels, we substituted ammonium with glutamate as the nitrogen source (YGD). Due to the direct deamination of glutamate to produce αKG and ammonium, YGD grown cells accumulated high levels of αKG and exhibited a 8-fold increase in the bulk αKG/succinate ratio compared to YAD (Fig. 2b, Supplementary Data Fig. 4). In association with this increase in the αKG/succinate ratio, we found that bulk H3K4me3 levels in WT cells grown in YGD were reduced compared with those grown in YAD. Notably, in YGD-grown cells, *jhd2∆* caused a 2.3-fold increase in H3K4me3 levels relative to WT, suggesting that Jhd2 demethylated H3K4 in response to an elevated αKG/succinate ratio (Fig. 2a).

To evaluate the global gene expression regulatory roles of *JHD2* in cells with nutritionally manipulated αKG/succinate, we performed RNA-Sequencing (RNAseq) experiments of WT and *jhd2∆* cells grown in YAD and YGD. Deletion of *JHD2* caused a modest gene expression defect in YAD, impacting the abundance of 213 mRNAs, although these genes did not fall into any significant GO term categories (q-value < 0.05, fold change >1.4, Supplementary Table 2). In YGD-grown cells, despite causing a significant increase in bulk H3K4me3 abundance, deletion of *JHD2* did not impact a substantially increased number of mRNAs compared with YAD with only 255 differing in abundance (q-value < 0.05, fold change >1.4, Supplementary Table 3). In contrast to in YAD however, GO term analysis revealed that the *JHD2*-regulated mRNAs in YGD did fall into specific categories: *JHD2*-activated mRNAs were significantly enriched for ribosomal protein encoding genes (*p* < 3e-09), while the *JHD2*-repressed mRNAs encode sequence specific DNA binding proteins (*p* < 4e-10, Supplementary Table 3). Extending findings from ES cells, our results support the hypothesis that JARID-family demethylases can respond to an increased αKG/succinate ratio to accomplish a substantial degree of bulk H3K4me3 demethylation. As abolishment of H3K4me itself causes limited gene expression consequences in fermenting yeast cells, the relatively minor impact that *JHD2* had on mRNA accumulation under these conditions is perhaps to be expected^1^,^15^.

### *JHD2* controls mitochondrial respiration through H3K4me

Unlike our results from YPA grown cells, *JHD2* did not impact mitochondrial transcripts in YAD or YGD media. In budding yeast, an abundance of glucose leads to mitochondrial repression and preferential fermentation of glycolytically produced pyruvate to ethanol, a phenomenon similar to the Warburg and Crabtree effects originally described in cancer cells^16^. The absence of any *jhd2∆* mitochondrial transcript signature in YAD or YGD (or YPD) may thus be due to glucose repression overriding any detectable role for *JHD2* in regulating these genes. Indeed, like in YPD, we found that fermenting cells grown in YAD or YGD media exhibited negligible accumulation of mitochondrial biomass and low respiratory rates compared with cells grown in YPA (Supplementary Data Fig. 2 and data not shown). To circumvent the Crabtree effect and interrogate *JHD2* function in respiratory cells grown in synthetic media, we substituted glucose with the carbon raffinose in Y media (YAR). YAR grown cells accumulated mitochondrial biomass, and exhibited 3-fold increased mitochondrial respiration compared with YAD (Supplementary Data Fig. 2). Moreover, RNAseq profiling confirmed that YAR grown cells accumulated mRNAs encoding components of the ETC (*p* < 1e-14, Supplementary Table 4).

Western blotting experiments revealed that H3K4me3 levels were increased 1.8-fold in *jhd2∆* cells grown in YAR, suggesting that Jhd2 activity was enhanced in YAR (Fig. 2c). However, using LC-MS we found that YAR grown cells exhibited a ratio of αKG/succinate indistinguishable from cells grown in YAD, where we did not find evidence for *JHD2*-dependent H3K4 demethylation (Fig. 2b, Supplementary Data Fig. 4). While we do not know how the YAR metabolic context may cause enhanced Jhd2 activity, other metabolites both known and unknown could impact Jhd2 function. One such metabolite, fumarate, has been suggested to inhibit the activity of a Jumonji-family demethylase^6^,^17^. Our LC-MS measurements revealed that fumarate levels were increased in YAR compared with YAD however, arguing against a role for reduced fumarate levels leading to Jhd2 activation in YAR (Supplementary Data Fig. 4). Importantly, our experiments measured bulk metabolites and therefore do not rule out the possibility that subcellular accumulation of αKG, succinate, and/or fumarate might impact Jhd2 activity.

We next used western blotting to determine if increased Jhd2 protein levels in YAR could explain the enhanced *JHD2*-dependent H3K4 demethylation we observed in this nutritional context. Curiously, we found that both Jhd2 and Set1 protein levels were reduced in cells grown in YAR media compared with in YAD, with Set1 levels being much more markedly reduced than Jhd2 (Supplementary Data Fig. 5). These results reveal that while Jhd2 levels in the cell were not increased in YAR, the ratio of Jhd2 to Set1 appeared to be markedly increased, perhaps explaining the greater role for *JHD2* in controlling bulk H3K4me levels in YAR. An alternative, though not mutually exclusive potential mechanism explaining the role of *JHD2* in YAR may relate to histone acetylation, which has been shown to have a negative impact on Jhd2 demethlyation^11^. We found that numerous histone lysine residues were substantially hypo-acetylated in YAR compared with YAD, perhaps providing a more permissive chromatin context and enabling Jhd2 demethylation of H3K4 (Fig. 2d)^11^.

Our findings identify two metabolic contexts that lead to enhanced Jhd2-mediated demethylation of H3K4me3, one in which Jhd2 activity is associated with an elevated αKG/succinate ratio (YGD), and another which cannot be explained by the accumulation of these metabolites (YAR). Whatever mechanism leads to enhanced *JHD2* function in YAR, *jhd2∆* nevertheless caused a bulk increase in H3K4me3 in these cells. To determine the gene expression consequences of *jhd2∆* in this nutritional context, we performed RNA sequencing experiments to measure mRNA transcripts from YAR grown cells. In stark contrast to its role in YAD or YGD, *jhd2∆* had a massive impact on gene expression in YAR: we identified 1193 genes that were significantly mis-regulated in *jhd2∆* mutants grown in YAR compared with WT controls (q-value < 0.05, fold change >1.4, Supplementary Table 5). As we observed in YPA, mRNAs encoding components of the mitochondrial electron transport chain (ETC) were highly significant targets of *JHD2*-repression in YAR (*p* < 2.6e-12, Supplementary Table 5).

To further explore the gene regulatory roles of *JHD2* in YAR, we analyzed strains carrying plasmids that over-express *JHD2* using a strong constitutive *ADH1* promoter. Consistent with a previous report^13^, *JHD2* over-expression, but not over-expression of an enzymatically dead version of *JHD2*, caused decreased H3K4me3 abundance in both YAR and YAD (Fig. 2e and data not shown). Despite the reduced H3K4me3 caused by *JHD2* overexpression in YAD, RNAseq profiling revealed that *JHD2* overexpression had a negligible consequence on the accumulation of mRNAs in YAD, with only 33 gene transcripts significantly altered compared with an empty vector control strain (q-value < 0.05, fold change >1.4, Supplementary Table 6). In stark contrast, *JHD2* overexpression in YAR impacted the abundance of 1240 mRNAs (q-value < 0.05, fold change >1.4, Supplementary Table 7). The majority of these transcripts were reduced in abundance in *JHD2* overexpressing cells, with mRNAs encoding ETC components showing a highly significant enrichment (*p* < 2e-14, Supplementary Table 7).

To comparatively analyze our RNAseq data, we identified 316 mRNAs that were significantly altered in abundance in both *jhd2∆* and *JHD2* overexpressing cells grown in YAR and subjected their relative abundance values to k-means clustering. We identified 4 clusters, labeled I - IV (Fig. 2f and Supplementary Table 8). The two largest clusters, I and III, were dominated by transcripts whose levels were opposingly affected in *jhd2∆* and *JHD2* overexpressing cells, as would be expected if their gene expression was modulated through H3K4me3. In these clusters, the abundance of the mRNAs was increased in *jhd2∆* and decreased in *JHD2* overexpressing cells, and their respective genes were highly enriched in the GO-term categories glycolysis and gluconeogenesis (cluster I, *p* < 2.5e-10) and mitochondrial ETC (cluster III, *p* < 1e-14, Supplementary Table 8). Interestingly, the mRNAs in clusters II and IV were similarly affected in both *jhd2∆* and *JHD2* overexpressing cells. The cluster II mRNAs were increased in abundance in both *jhd2∆* and *JHD2* overexpressing cells and were highly enriched for genes involved in transmembrane transport (*p* < 2e-10) while Cluster IV mRNAs were decreased in abundance in both genetic conditions and encoded proteins involved in mitochondrial translation (*p* < 2.3e-07) (Fig. 2f and Supplementary Table 8). It is difficult to explain the curious characteristics of cluster II and IV genes with a simple model invoking H3K4me3 accumulation. We thus think more complex models involving dynamics in H3K4me or non-enzymatic functions and/or non-histone substrates of Jhd2 may underlie the regulation of these genes.

**Figure 3:**
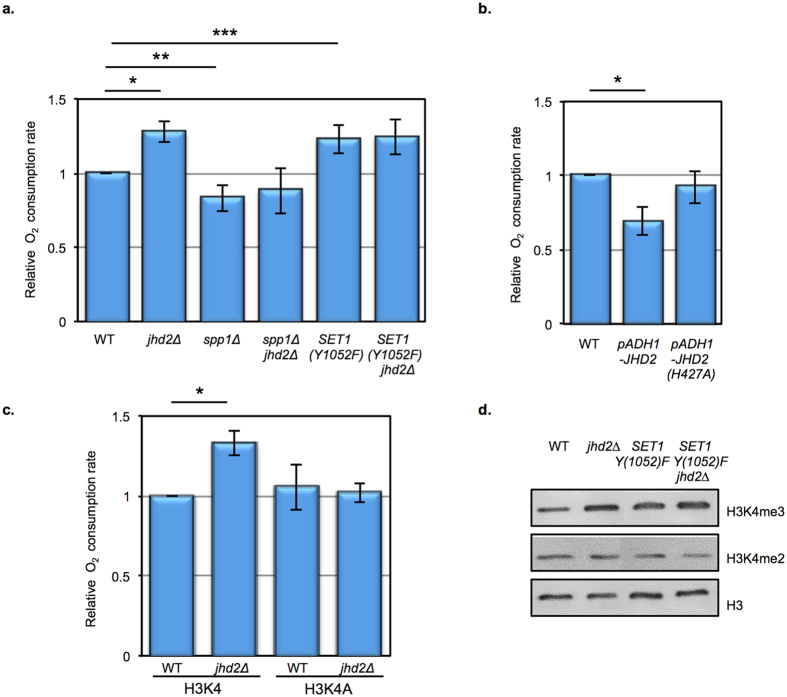
*JHD2* controls mitochondrial respiration through H3K4me3. Oxygen consumption rates were measured in the indicated genotypes grown in YAR (**a** and **b**) or YPA (**c**). n = at least 3 for all experiments, and error bars depict 1 s.d. Significance as calculated by a two-tailed student’s t-test is shown. (**a**) Respiration rates in strains of the indicated genotypes are shown (MMY5020, MMY5023, MSY840, MSY838, MMY5021, and MMY5024). *p = 0.0002, **p = 0.01, and ***p = 0.004. (**b**) Respiration rates in strains of the indicated genotypes are shown (MMY5087, MMY5086, and MMY5085). *p = 0.004. (**c**) Respiration rates in strains of the indicated genotypes are shown (MSY883, MSY889, MSY885, and MSY891). *p = 0.002. (**d**) Western blot detection of H3K4me3, H3K4me2, and pan-H3 are shown from extracts of cells grown in YAR and of the indicated genotypes and *SET1(Y1052F*) plasmids (MMY5020, MMY5023, MMY5021, and MMY5024).

Our RNAseq studies in both *jhd2∆* and *JHD2*-overexpressing strains showed that *JHD2* repressed the accumulation of mRNAs encoding mitochondrial proteins and ETC components. We therefore measured mitochondrial respiration in YAR and confirmed that, like in YPA media, *jhd2∆* caused a hyper-respiratory phenotype (Fig. 3a). Moreover, *JHD2* over-expression, but not that of an enzymatically dead allele of *JHD2*, caused decreased respiration in YAR grown cells (Fig. 3b). These results suggest that *JHD2* impacts mitochondrial respiration through its control of the gene expression program via modulation of H3K4me3.

If *JHD2* impacts mitochondrial respiration through H3K4me3, then reversal of the H3K4me3 defects should revert the *jhd2∆* hyper-respiratory phenotype. To test this, we first engineered strains in which histone H3 function was provided by plasmids expressing either WT H3 or an allele of H3 encoding an unmethylatable alanine residue in place of H3K4 (H3K4A). Using this genetic system, we found that the hyper-respiratory phenotype exhibited by *jhd2∆* in YPA was dependent on H3K4 (Fig. 3c). To investigate the role of H3K4me3 specifically, we engineered strains with deletion alleles of *SPP1*, which encodes a subunit of the Set1-complex required for H3K4me3 accumulation, but not for that of mono- or di-methylated species of H3K4 (Fig. 2c)^18^. Deletion of *SPP1* caused a hypo-respiratory phenotype in YAR and *spp1∆* was epistatic to *jhd2∆* for respiration, further supporting a model whereby increased H3K4me3 caused the hyper-respiration exhibited by *jhd2∆* cells (Fig. 3a).

To further test the model that H3K4me3 levels modulate mitochondrial activity as opposed to some other function of *JHD2*, we interrogated a strain expressing *SET1(Y1052F)*, a hyperactive allele of *SET1* known to cause increased accumulation of H3K4me3 (Fig. 3d)^19^. In YAR media, *SET1(Y1052F*) caused a hyper-respiratory phenotype that was indistinguishable from that of *jhd2∆* (Fig. 3a). Interestingly, *SET1(Y1052F*) did not synergize with *jhd2∆* for bulk H3K4me3 accumulation or for respiration, suggesting that *JHD2* and *SET1(Y1052F*) globally control the same H3K4me3 targets to affect mitochondrial control (Fig. 3a and d).

**Figure 4:**
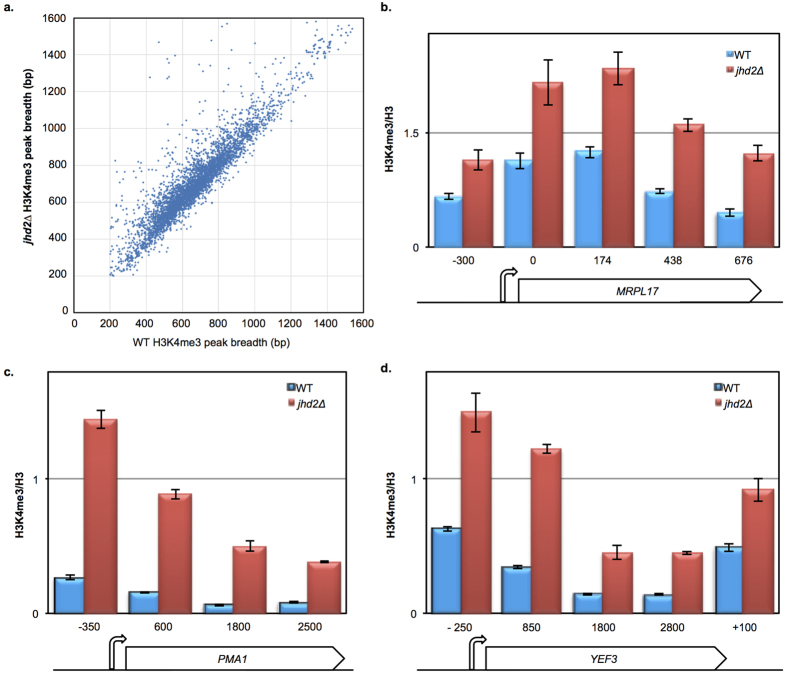
*JHD2* restricts accumulation of H3K4me3 across genes. (**a**) A clustergram depicting peak breadth is shown. Each point represents the breadth of corresponding H3K4me3 peaks identified using ChIP-seq in *jhd2∆* (y-axis) or WT (x-axis) from YAR grown cells (MSY723, MSY724). (**b–d**) ChIP-qPCR was used to quantify the abundance of H3K4me3 relative to pan-H3 across the indicated genes (MSY723, MSY724). PCR amplicon locations across these genes are indicated below. Error bars represent 1 s.d. of technical replicates.

The bulk accumulation of H3K4me3 we observed in *jhd2∆* cells grown in YAR suggested a genome-wide impact on H3K4me3 accumulation rather than at specific genomic loci, which we initially attempted to characterize using chromatin immunoprecipitation followed by DNA sequencing (ChIP-Seq). Using ChIP-Seq, we found that *jhd2∆* caused an increase in the breadth of H3K4me3 domains across many genes in YAR, although the genes that exhibited increased breadth did not correlate with mRNAs whose abundance was regulated by *JHD2* (Fig. 4a and data not shown). A Wilcoxon signed rank test confirmed that this increased breadth is greater than would be predicted by chance (Supplementary Data Fig. 6).

The analysis of ChIP-Seq data requires normalizations that equalize total H3K4me3 signals between WT and *jhd2∆*. We thus suspect that these normalization steps obscured our ability to detect genome-wide H3K4me3 accumulation caused by *jhd2∆*, resulting in only a few regions exhibiting a increased breadth phenotype^20^. This interpretation of our ChIP-Seq data makes the prediction that conventional ChIP followed by PCR quantification (ChIP-qPCR) interrogation of genes that do not exhibit a breadth phenotype should reveal that these regions in fact have elevated H3K4me3 levels in *jhd2∆*.

To test this, we chose three genes that did not exhibit a breadth phenotype in our ChIP-Seq data: the mitochondrial gene *MRPL17*, as well as the well-studied and highly expressed genes *PMA1* and *YEF3*. ChIP-qPCR revealed that *jhd2∆* caused a marked increase in H3K4me3 spanning these loci (Fig. 4b–d). Analysis of a biological replicate confirmed these findings (Supplementary Data Fig. 7). Collectively, we interpret our findings as indicative of a general increase in H3K4me3 spanning the entire transcribed genome in *jhd2∆* cells grown in YAR media, across mitochondrial-encoding and non-mitochondrial encoding genes alike. As the increased H3K4me3 at these genes evaded detection by ChIP-Seq, the increased breadth phenotype we describe using ChIP-Seq seems likely to only superficially reflect H3K4me3 accumulation in *jhd2∆*. More specifically, we think the increased H3K4me3 breadth we observed may reflect a genome-wide accumulation of H3K4me3, which can only be detected along the edges of certain genomic regions owing to artifactual normalization in the absence of externally added “spike-in” controls^20^. This insight may have ramifications for the interpretation of other investigations of H3K4me3 breadth using ChIP-Seq data^21^,^22^. As *jhd2∆* caused increased H3K4me3 in both YGD and YAR grown cells, the broad gene expression defects we observe in in YAR, but not in YGD, seem likely to reflect contextual specific roles for H3K4me3 in respiring vs. fermenting cells.

### Cellular proliferation is restrained by *JHD2*

**Figure 5:**
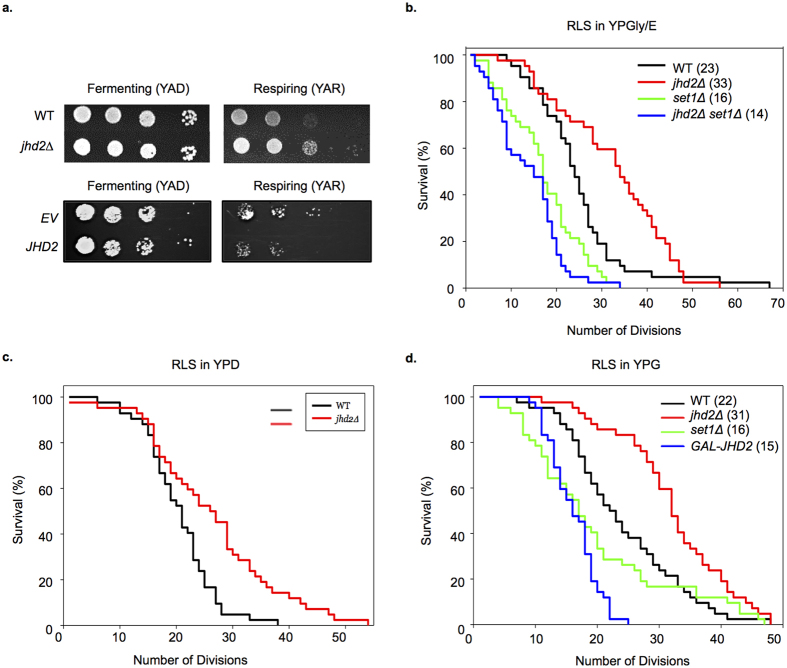
*JHD2* restricts cell proliferation. (**a**) Colony growth of cells of the indicated genotypes (MSY723, MSY724, MMY5087, and MMY5086) grown under fermentative (YAD) or respiratory conditions (YAR) are shown. (**b**) Replicative lifespans were measured for WT (MSY576), *jhd2Δ* (MSY577), *set1Δ* (MSY578), and *set1Δ jhd2Δ* (MSY579) cells grown under respiratory conditions with glycerol ethanol rich media (YPGE). (**c**) Replicative lifespans were measured for WT (MSY576) and *jhd2Δ* (MSY577) cells grown in YPD media. (**d**) Replicative lifespans were measured for isogenic WT (MSY576), *jhd2Δ* (MSY577), *set1Δ* (MSY578), and *P*_*GAL*_*-JHD2* (MSY727) cells grown in galactose rich media. The median lifespan (in generations) is indicated in brackets for each genotype.

Perturbations of *JHD2* orthologs impact proliferative properties of human and insect cells, foster tumor persistence, and promote drug tolerance of cancer cells^23^,^24^,^25^,^26^,^27^,^28^,^29^,^30^,^31^. Intriguingly, the persistence of JARID-overexpressing cancer cells is dependent on ETC function^32^. To determine if the ETC regulatory roles of *JHD2* were associated with proliferative phenotypes in yeast, we assessed colony growth of *jhd2∆* and *JHD2*-overexpressing cells on media with fermentable and non-fermentable carbon sources. We found that, while neither *jhd2∆* nor *JHD2*-overexpression caused a colony growth phenotype on cells grown under fermentative conditions (YAD), they did impact colony growth from cells grown under respiratory conditions (YAR): *jhd2∆* permitted enhanced growth and *JHD2*-overexpression caused reduced colony growth (Fig. 5a).

To examine the proliferative role of *JHD2* using a single cell assay, we determined the number of mitotic divisions that individual cells with manipulated *JHD2* activity were capable of using the replicative lifespan assay. In this assay, the “lifespans” of individual yeast cells are determined by counting the number of daughter cells it produces before the mother cell eventually senesces. We found that *jhd2∆* caused a significant extension of yeast lifespan on rich media containing the non-fermentable carbons glycerol and ethanol (YPGE) (Fig. 5b and Supplementary Data Table 1). Consistent with a previous report that assessed lifespans of *set1∆* on glucose containing media, we found that *set1∆* cells were short-lived on YPGE (Fig. 5b)^33^. Moreover, *set1∆* was epistatic to *jhd2∆* for lifespan extension, consistent with the conclusion that *JHD2* limited lifespan through H3K4 demethylation (Fig. 5b). Somewhat surprisingly, *jhd2∆* also caused lifespan extension on YPD, a consequence that may be attributable to the dramatic changes in cellular physiology and mitochondrial function that occur in aging yeast cells grown under these conditions^34^ (Fig. 5c). We next utilized a strain engineered to express *JHD2* under the control of a *GAL1* promoter, which directs high-level transcription in media containing galactose as the sole carbon source, and found that *P*_*GAL*_*-JHD2* caused a dramatic shortening of lifespan, comparable with that caused by *set1∆* (Fig. 5d). These results suggest that *JHD2* restrains the proliferative capacity of individual yeast cells in a *SET1*-dependent manner.

## Discussion

Similar to the archetype of Sirtuin lysine deacetylases responding to the ratio of NAD/NADH in the cell, intracellular pools of αKG and other metabolites have been implicated in the regulation of Jumonji histone demethylases^9^,^35^. We have described here further evidence for this: in fermenting cells, nutritional manipulations that elevate the αKG/succinate ratio were associated with *JHD2*-dependent reductions in bulk H3K4me3. Like in human embryonic stem cells, catabolism of glutamate is responsible for the elevated αKG in these cells. Although not further elaborated on here, our results show that in cells with elevated αKG levels, *JHD2* promoted the expression of a large number of ribosomal protein encoding genes, a function perhaps related to previous findings implicating *JHD2* in nucleolar processes^36^. Our studies underscore budding yeast as a powerful model to address the emerging issue of how αKG accumulation leads to changes in gene expression changes through regulated histone demethylation.

We also identified respiring cells as a context in which *JHD2*-dependent reductions in H3K4me3 occur, although this function was manifested in the absence an elevated αKG/succinate ratio. Indeed, in contrast to cells grown in YAD, cells grown in YAR exhibited *JHD2*-dependent reductions in global H3K4me3 accumulation despite having bulk levels of both αKG and succinate that were indistinguishable from those grown in YAD. Of the numerous possible mechanisms underlying this finding, we found that YAR grown cells also possessed reduced levels of varied histone acetylations, which have been shown to have an inhibitory impact on Jhd2-mediated H3K4 demethylation^11^.

Perhaps the most intriguing aspect of our findings concerns the identification of mitochondrial regulation as a target of *JHD2*, what appears to be an emerging role of this ancient gene family. Similarly to our work, mitochondrial respiration has been identified as a target of JARID1A repression in mouse embryonic fibroblasts, where JARID1A knockdown restores the ability of cells lacking Retinoblastoma tumor suppressor protein function to differentiate in a manner that is dependent on its demethylase activity^37^. In a finding that is superficially at odds with our results and those from human cells, in *Drosophila melanogaster*, Lid, the sole JARID ortholog present in this organism, functions to *activate* mitochondrial genes in a manner that is independent of its enzymatic activity^38^. We point out that these studies were performed using whole flies, and thus describe the net role of Lid in what are overwhelmingly terminally differentiated and non-proliferative cells. This is in contrast to both our studies and the human studies, which interrogated proliferating cells. Thus, JARID family proteins may opposingly regulate mitochondrial activity in proliferative vs. differentiated cells using alternative mechanisms. Our findings using proliferative yeast suggest that *JHD2*’s regulatory impact on mitochondria occur through its control of global H3K4me3 levels, an interpretation that is in good agreement with what has been shown in human cells.

There are two outstanding questions raised by these findings: 1. How is it that JARID control of H3K4me impacts genes encoding mitochondrial proteins in proliferating cells? and 2. Does our identification of mitochondria as a target of *JHD2* control in yeast reflect an ancestral function for this gene family in mediating mitochondrial feedback control? Both of these questions can perhaps be addressed by determining the molecular mechanisms of mitochondrial gene regulation by JARID orthologs and then asking if these mechanisms themselves are conserved. As a prominent model system for studies of chromatin biology, yeast is well poised to address the critical question of mechanism, which may thus have both biomedically and evolutionary relevant implications. Perhaps related to this, our previous studies showed that *JHD2* opposes spore differentiation, a role that is intriguingly similar to what has been shown for JARID family proteins during human stem cell differentiation^2^,^23^,^26^,^27^,^37^,^39^. It will be interesting to determine how the mitochondrial regulatory roles of *JHD2* impinge upon its role in spore differentiation, and whether these mechanisms also underlie the roles of JARID proteins in differentiating stem cells. Finally, our identification of Cox2, a protein encoded by the mitochondrial genome, as a target of *JHD2* repression in the absence of any *COX2* mRNA regulation suggests that Jhd2 may have additional substrates that impact mitochondrial function beyond H3K4.

## Methods

### Yeast genetics and growth conditions

Standard yeast genetic methods were used for construction of all strains. Yeast strains and plasmids used in this study are listed in Supplementary Data Tables 2 and 3. All strains were constructed through genetic crosses followed by dissections of either SK1 or the BY4742 backgrounds. Rich YP media consisted of 1% yeast extract, 2% peptone, and 2% of the indicated carbon source (glucose, galactose, or potassium acetate). Synthetic defined minimal YNB media (Multicell Wisent) contained 2% carbon (glucose or raffinose) and 5 g/L of ammonium sulfate or 2 g/L of monosodium glutamate as indicated. For mass spectrometric experiments, a mixture of ammonium acetate and ammonium sulfate was used in place of ammonium sulfate. Unless noted, for all experiments, cells were grown to uniform O.D._600_ between 0.3–0.6 using conditions that ensured at least 5 doublings in logarithmic phase growth prior to analysis.

### Western blotting

Protein extracts were prepared and western blotting performed as described previously^2^ following normalization for total protein concentration using an RC/DC assay (BioRad). Equal amounts of protein were electrophoresed on 12% SDS-PAGE gels, and transferred onto Amersham Hybond-P membranes (GE). Primary antibodies for histone and mitochondrial protein westerns were purchased from Abcam: ab1791 (pan-H3), ab8580 (H3K4me3), ab7766 (H3K4me2), and ab10326 (Por1). Tom Fox provided a Cox2 antibody. TAP-Jhd2 and TAP-Set1 were detected using anti-peroxidase (Abcam ab21867). All blots were scanned with a Typhoon scanner as described^2^. Band intensities were quantified using ImageJ 1.37 v software. For most western blotting experiments, a minimum of 3 biological replicates was analyzed to enable statistical evaluations as indicated in the figure legends.

### Metabolite measurements

The prototrophic S288C strain RCY308 was cultured and metabolites were prepared according to established procedures^40^. Yeast metabolite extracts were reconstituted to a concentration of 0.6 OD_600_ of yeast in 40 uL of HPLC-grade H_2_O by vortexing for 1 minute. The reconstituted extract was combined with an equal volume of isotopically labeled material as an internal standard. LC-MS analysis was performed using an Agilent Technologies 6540 qTOF spectrometer equipped with a Jet Spray ESI source operated in negative ionization mode. Reverse phase separation as performed using a 2.1 mm × 100 mm Waters HSS-T3 C18 column with 1.8 um packing. Metabolite levels are presented as integrated areas relative to co-eluting isotopic reference. To ensure statistical power, a minimum of 4 biological replicates was performed for each nutritional context.

### Oxygen consumption assays

Oxygen consumption of cells in suspension was measured using an oxygen Clark-type electrode (Strathkelvin) as previously described^2^. For basal oxygen consumption measurements, cells were washed once and measured in their media. Oxygen consumption rate was measured in units of nmol atoms (natoms) of oxygen per minute per one million cells (natom/min/M). For biological replicate comparison, each sample was normalized to its corresponding WT control and these ratios were averaged. For all respiration experiments, a minimum of 3 biological replicates was performed to enable statistical analysis as indicated in the figure legends.

### Microarray transcript profiling and RT-qPCR analysis

RNA was prepared using the hot acid phenol method from quadruplicate mmy718 (WT) and mmy1879 (*jhd2∆*) cultures grown to mid-logarithmic growth phase (O.D._600_ = 0.8) in YPA. cDNA was prepared and analyzed on custom Agilent microarrays using standard methods at the Ontario Cancer Institute Genomics Centre microarray facility. Normalization, and data analysis was performed as described previously^2^. For RT-qPCR, RNA was prepared from 4 independent biological cultures of WT and *jhd2∆* and cDNA was prepared as described previously using random priming^2^. Quantitative PCR quantification of cDNA was performed as described previously^2^, and transcript levels for each primer pair tested were normalized to the reference transcript *SCR1*. Average expression levels were normalized to WT and standard deviations were obtained across the four biological replicates.

### RNA-sequencing

Replicate cultures of MSY723 (WT) and MSY724 (*jhd2∆*) were serially outgrown to O.D._600_ = 0.5. For the *jhd2∆* vs WT YAR data set, 2 biological replicates were analyzed. For all others, 3 biological replicates were used. Pellets of 1 O.D._600_ were snap frozen in liquid nitrogen and RNA was prepared as described above following addition of 10 uL of 1:100 dilution of ERCC92 Mix 1 (Ambion/Life) to each cell pellet as an external spike-in control. Total RNA was DNase treated, and purified with Qiagen RNeasy columns. Ribosomal RNA was depleted using an Illumina Yeast RiboZero rRNA depletion kit (MRZY1306), followed by RNA fragmentation and priming with Illumina TruSeq Stranded mRNA library synthesis (CAT RS-122-2101). All protocols were performed according to manufacturer’s directions. Single read sequencing was completed on the Illumina HiSeq2500 platform with Version 4 reagents at the Donnelly Sequencing Centre. Single-end reads of 51 bp in length were acquired with a total of 240–250 million reads per lane.

FASTQ files were checked for sequencing quality using FastQC (Version 0.10.1 2011). Sequences were aligned to the yeast genome (build 3.1) using TopHat (version 2.0.12), utilizing Bowtie2 aligner (version 2.1.0). Files from TopHat were processed using Cufflinks (version 2.1.1) to yield transcript annotations and FPKM values (fragments per kilo base transcript per million reads). Differential transcript abundance analysis was performed using Cuffdiff (version 2.1.1) with transcript identification guided by a gtf file specifying the location of yeast transcripts compiled from SGD (Saccharomyces Genome Database). K-means clustering was performed using Cluster 3.0 and visualized using Java TreeView (1.1.6r4). For the heat map shown in Fig. 2f, the maximum number of clusters for which we could obtain a solution 100/100 times was four. We therefore proceeded with analysis of these four clusters.

### ChIP-seq and ChIP-qPCR

YAR cultures of MSY723 (WT) and MSY724 (*jhd2∆*) were serially outgrown to O.D._600_ = 0.5. Chromatin immunoprecipitation of H3K4me3 and H3 was performed as described previously^2^. Samples were sonicated on the high setting of a Diagenode Bioruptor for 30 second cycles with 30 second breaks in between. Total sonication time was 15 minutes and shearing to an average fragment length of 500 base pairs was confirmed by agarose gel electrophoresis. For ChIP-seq, sequencing libraries for IP and WCE were prepared at the Donnelly Sequencing Centre using standard methods. Single read sequencing was completed on the Illumina HiSeq2500 platform with Version 4 reagents. Single-end reads of 51 bp in length were acquired with ~14 million reads per sample, equivalent to 60-fold genome coverage. Read quality was assessed using FastQC (Babraham Bioinformatics). Fastq reads were aligned to the yeast reference genome (downloaded from iGenomes, Illumina) using bowtie2 (version 2.2.3) with standard parameters. Reads had >90% alignment rate. Data was processed using SAMtools, Picard and IGV^41^. Depth of coverage was determined genome-wide using Genome Analysis Toolkit (GATK, Broad Institute) DepthOfCoverage.jar tool. H3K4me3 peak and peak breadth calling was done with MACS2 software, with the broad region calling option turned off^42^. 4509 gene-associated peaks were identified in both WT and *jhd2∆*. To test the significance in the difference in H3K4me3 peak breadths over genes between WT and *jhd2∆*, Wilcoxon signed rank tests were performed using SigmaPlot. For ChIP-qPCR, immunoprecipitation (IP) and whole cell extract (WCE) fractions were analyzed as described previously^2^.

### Replicative lifespan analysis

Cells of the indicated genotypes were patched lightly onto rich media plates with the indicated carbon sources. At least 42 cells were arrayed out and virgin daughter cells were picked for analysis. Kaplan-Meier curves were used to plot the data as percent survival.

### Statistics

Two-tailed Student’s T-tests were used to analyze significance between respiration rates of different strains as well as differences in protein levels and gene expression variance. Determination of lifespan significance was performed using a one tailed Mann-Whitney U test. Funspec^43^ was used for GO-term enrichment analysis of gene expression data sets.

#### Fluorescence microscopy

Images were generated from live cells. Three-dimensional images were reconstructed from 0.5 μm Z-stacks captured using a Leica DMI6000 microscope equipped with a WaveFX spinning disc confocal system. Volocity High Performance 3-D 4-D imaging software was used to reconstruct 3D images.

### Data availability

RNA-seq, microarray, and ChIP-seq data generated in this paper have been deposited at the NCBI Gene Expression Omnibus (GEO) under accession number GSE85322.

## Additional Information

**How to cite this article**: Soloveychik, M. *et al*. Mitochondrial control through nutritionally regulated global histone H3 lysine-4 demethylation. *Sci. Rep.* **6**, 37942; doi: 10.1038/srep37942 (2016).

**Publisher's note:** Springer Nature remains neutral with regard to jurisdictional claims in published maps and institutional affiliations.

## Supplementary Material

Supplementary Data 1

Supplementary Data 2

Supplementary Data 3

Supplementary Data 4

Supplementary Data 5

Supplementary Data 6

Supplementary Data 7

Supplementary Data 8

Supplementary Data
