# Supplementary material for: Mitochondrial control through nutritionally regulated global histone H3 lysine-4 demethylation
Source: Sci Rep. 2016 Nov 29;6:37942. doi: 10.1038/srep37942 (PMC5126570; doi:10.1038/srep37942)
Supplement: Supplementary Data [file srep37942-s9.pdf]

Mitochondrial control through nutritionally regulated global histone H3 lysine-4 demethylation

Maria Soloveychik[1], Mengshu Xu[1], Olga Zaslaver[1,2], Kwanyin Lee[1], Ashrut Narula[1], River Jiang[1], Adam Rosebrock[1,2], Amy Caudy[1,2], and Marc Meneghini[1]

[1] Department of Molecular Genetics, University of Toronto, ON, M5S 1A8, Canada

[2] Terrence Donnelly Centre for Cellular & Biomolecular Research, University of Toronto, ON, M5S 3E1, Canada

Supplementary Fig. 1

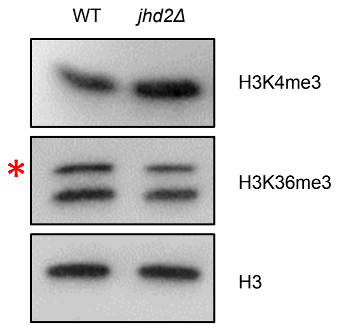

Supplementary Fig. 2

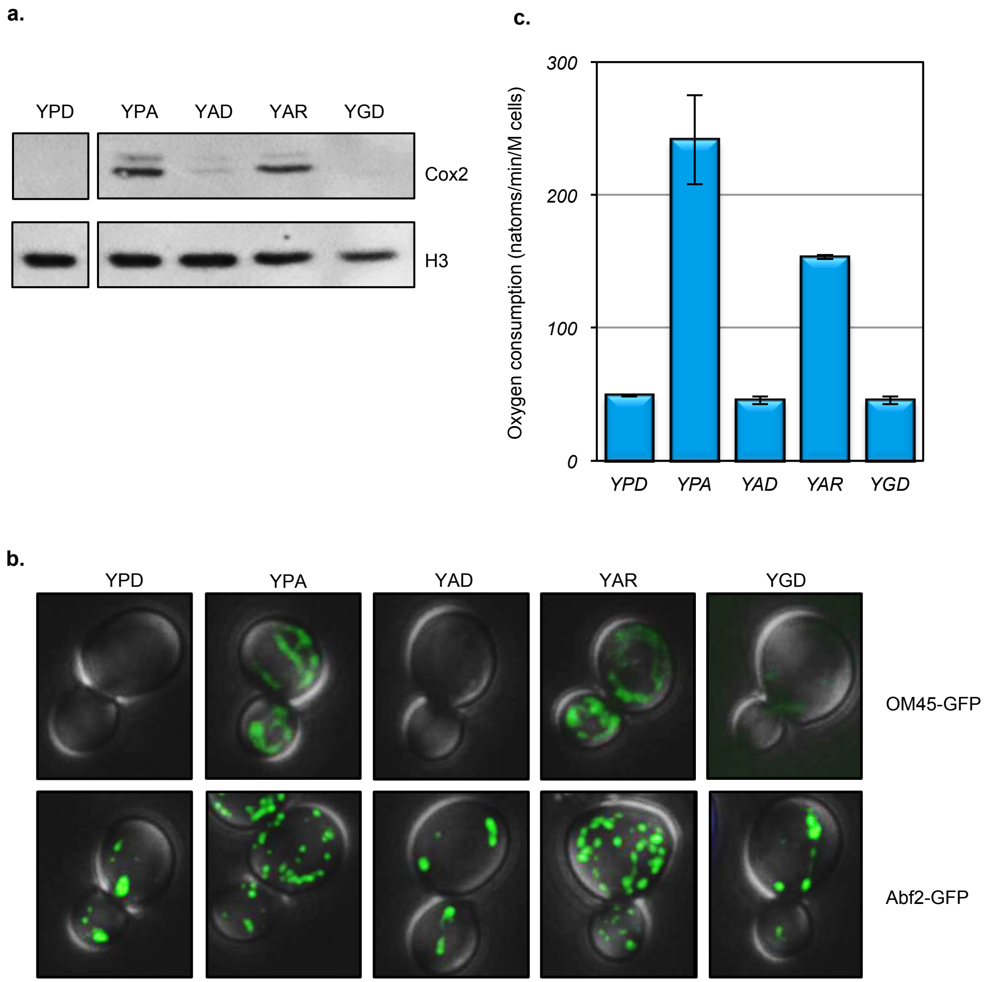

Supplementary Fig. 3

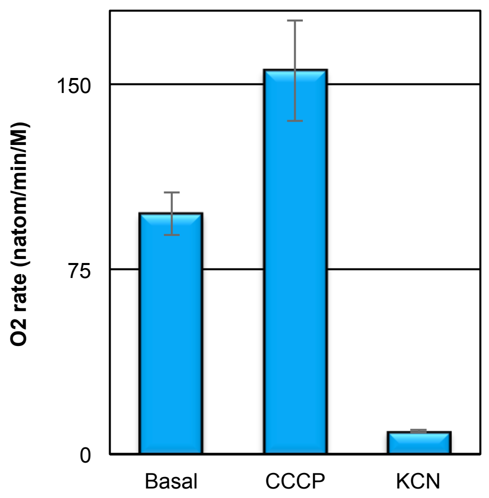

Supplementary Fig. 4

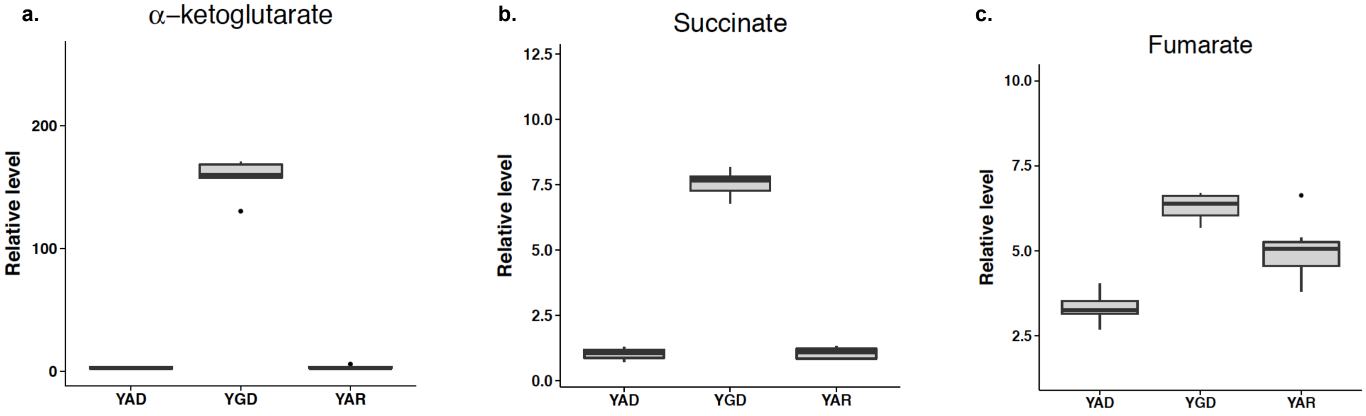

Supplementary Fig. 5

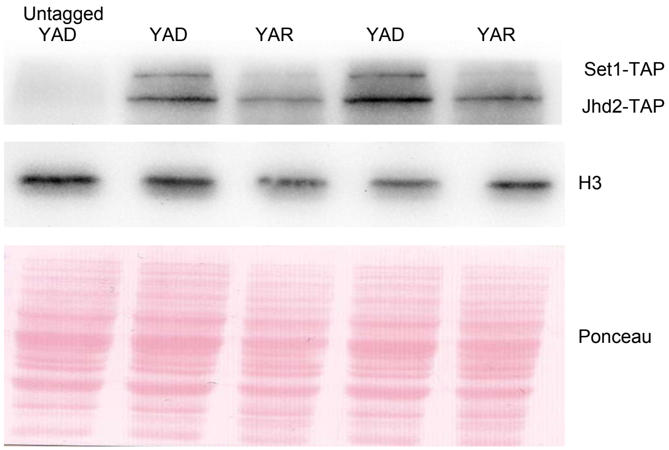

Supplementary Fig. 6

a.

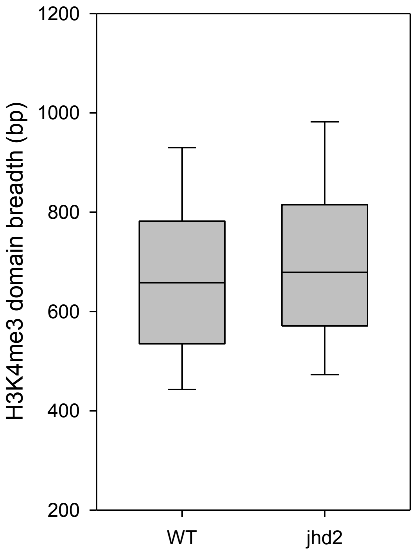

b.

**Group**	**N**	**Missing**	**Median**	**25%**	**75%**
WT	4509	0	658.000	535.000	782.000
*jhd2*Δ	4509	0	679.000	571.000	815.000

(*P* = <0.001)

Supplementary Fig. 7

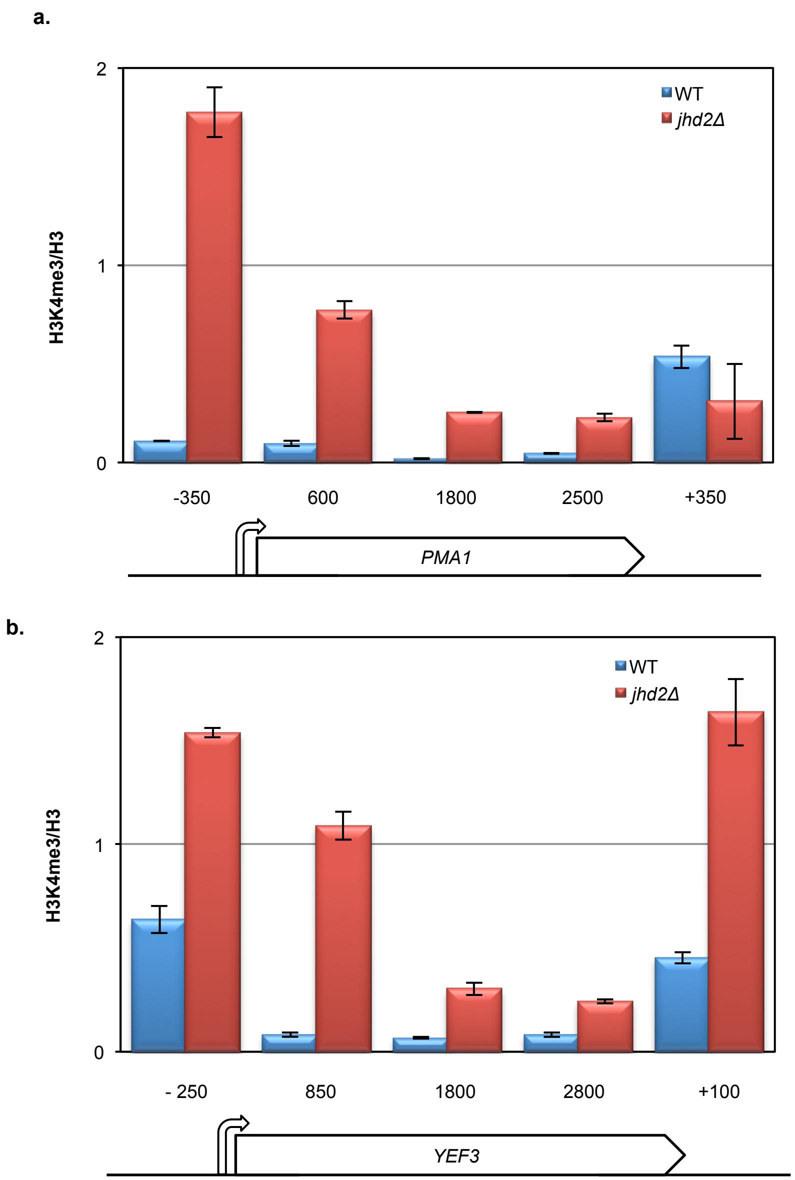

Supplementary Table 1

Carbon	Genotype 1	Genotype 2	*P* Value
Glycerol/ Ethanol	*WT*	*jhd2Δ*	*p = 6.5e-04*
	WT	*set1Δ*	*p = 1e-04*
	WT	*jhd2Δ set1Δ*	*p = 1e-06*
Glucose	*WT*	*jhd2Δ*	*p = 2.2e-03*
Galactose	*WT*	*jhd2Δ*	*p = 4e-06*
	WT	*set1Δ*	*p = 1.7e-03*
	WT	*GAL-JHD2*	*p = 1e-06*

Supplementary Table 2 | Strains used in the study

Strain	Background	Genotype	Source
msy576	BY4742	MATα his3 leu2 ura3 lys2	This study
msy577	BY4742	MATα his3 leu2 ura3 lys2 jhd2Δ::Hyg	This study
msy578	BY4742	MATα his3 leu2 ura3 lys2 set1Δ::Nat	This study
msy579	BY4742	MATα his3 leu2 ura3 lys2 jhd2Δ::Hyg set1Δ::Nat	This study
msy727	BY4742	MATa his3 leu2 ura3 lys2 GAL-JHD2::Nat	This study
msy723	BY4742	MATa prototrophic	This study
msy724	BY4742	MATa prototrophic jhd2Δ::Hyg	This study
msy840	BY4742	MATa prototrophic spp1Δ::Nat	This study
msy838	BY4742	MATa prototrophic jhd2Δ::Hyg spp1Δ::Nat	This study
msy1074	BY4742	MATa his3 JHD2-TAP::HIS3 SET1-TAP::HIS3	This study
mmy5085	BY4742	MATα leu2 + pGL99	This study
mmy5086	BY4742	MATα leu2 + pGL102	This study
mmy5087	BY4742	MATα leu2 + pAD4M	This study
mmy5020	BY4742	MATα leu2 set1Δ::Nat + mmb268	This study
mmy5021	BY4742	MATα leu2 set1Δ::Nat + mmb269	This study
mmy5023	BY4742	MATα leu2 set1Δ::Nat jhd2Δ::Hyg + mmb268	This study
mmy5024	BY4742	MATα leu2 set1Δ::Nat jhd2Δ::Hyg + mmb269	This study
msy883	BY4742	MATa his3 leu2 ura3 hht1-hhf1::KanMX hht2-hhf2::KanMX + mmb162	This study
msy885	BY4742	MATa his3 leu2 ura3 hht1-hhf1::KanMX hht2-hhf2::KanMX + mmb169	This study
msy889	BY4742	MATa his3 leu2 ura3 hht1-hhf1::KanMX hht2-hhf2::KanMX jhd2Δ::Hyg + mmb162	This study
msy891	BY4742	MATa his3 leu2 ura3 hht1-hhf1::KanMX hht2-hhf2::KanMX jhd2Δ::Hyg + mmb169	This study
msy757	BY4742	MATα prototrophic OM45-GFP::His3	This study
msy812	BY4742	MATa prototrophic ABF2-GFP::His3	This study
FY4	BY4742	MATa prototrophic	[42]
mmy720	SK1	MATa/x his3 leu2 ura3 trp1	This study
mmy881	SK1	MATx his3 leu2 ura3 trp1 jhd2::Kan	This study
mmy1499	SK1	MATx his3 leu2 ura3 trp1 gis1::Kan set1::Nat	This study
mmy1420	SK1	MATx his3 leu2 ura3 trp1 set2::Kan	This study

Supplementary Table 3 | Plasmids used in the study

Plasmid	Genotype	Source
mmb162	*CEN ARS TRP1 HHT1 HHT2 WT*	[43]
mmb169	*CEN ARS TRP1 HHT1 HHT2 H3K4A*	[43]
pGL99	*LEU2 2µm pADH1-JHD2(H427A)::FLAG*	[13]
pGL102	*LEU2 2µm pADH1-JHD2::FLAG*	[13]
pAD4M	*LEU2 2µm*	[13]
mmb268	*pRS315 CEN ARS LEU2 SET1*	[19]
mmb269	*pRS315 CEN ARS LEU2 SET1(Y1052F)*	[19]

Supplementary Figure 1 | Jhd2 is an H3K4me3 specific demethylase. a, Western blot detection of H3K4me3 and H3K36me3 levels in WT, *jhd2*Δ, *set1*Δ*,* or *set2*Δ and strains (MMY720, MMY881, MMY1499 or MMY1420).

Supplementary Figure 2 | Nutritional changes leading to mitochondrial biogenesis. a, Mitochondrial protein synthesis relative to H3 was determined using western blot detection of Cox2 and H3 in WT (MSY723) cells grown in the indicated nutritional sources. **b,** Shown are representative images of compacted z stacks of strains expressing the mitochondrial markers OM45-GFP (MSY757) and Abf2-GFP (MSY812) grown in the indicated media. **c,** Oxygen consumption was measured in the indicated media (MSY723), plotted is N=3, with 1 s.d. shown.

Supplementary Figure 3 | Oxygen consumption is due to mitochondrial respiration. WT cells (MSY723) were grown in YPA and oxygen consumption rates were determined for untreated cells or cells treated with 10 uM KCN or 8 uM CCCP. An average consumption arte for n =3 with 1 s.d. is shown.

Supplementary Figure 4 | Accumulation of metabolites in the various media. Relative LC-MS obtained metabolite levels in YAD, YGD, and YAR for **a,** αKG **b,** succinate and **c,** Fumarate for WT cells (FY4).

Supplementary Figure 5 | Jhd2 and Set1 accumulation in YAD and YAR.

A strain expressing both Jhd2-TAP and Set1-TAP (MSY1073) or a untagged strain (MSY723) were grown in the indicated media. Protein extracts from two biological replicates were examined by western blot for TAP or for H3. Ponceau S staining of the transferred proteins is shown.

Supplementary Figure 6 | H3K4me3 domain breadth is regulated by Jhd2. H3K4me3 domains were called from ChIP seq data using peak-calling software MACS2. H3K4me3 domains were attributed to genes based on overlap with the 5' coding region. The breadths of 4509 gene-associated H3K4me3 domains in WT and *jhd2Δ* cells are shown as a whisker plot. There is a significant difference in H3K4me3 domain breadths of genes in *jhd2Δ* compared to WT cells as determined by the Wilcoxon signed rank test ($P<0.0001$).

Supplementary Figure 7 | Genomic H3K4me3 levels are regulated by Jhd2 in vivo. ChIP-qPCR was used to quantify the abundance of H3K4me3 relative to pan-H3 across the indicated genes (MSY723, MSY724). PCR amplicon locations across these genes are indicated below. Error bars represent 1 s.d. of technical replicates.

Supplementary Table 1 | *JHD2* regulates yeast replicative lifespan. A Mann-Whitney U Test was used to measure the statistical significance between the differences of the indicated strains' lifespans from Figure 3.
